# Exogenous sex hormones, menstrual and reproductive history, and risk of non-melanoma skin cancer among women: a systematic literature review and meta-analysis

**DOI:** 10.1038/s41598-021-88077-y

**Published:** 2021-04-19

**Authors:** Saverio Caini, Simone Pietro De Angelis, Federica Corso, Carolina Fantini, Sara Raimondi, Laura Pala, Ignazio Stanganelli, Vincenzo de Giorgi, Sara Gandini

**Affiliations:** 1 Cancer Risk Factors and Lifestyle Epidemiology Unit, Institute for Cancer Research, Prevention and Clinical Network (ISPRO), Via Cosimo il Vecchio 2, 50141 Florence, Italy; 2 Department of Experimental Oncology, European Institute of Oncology (IEO), IRCCS, Milan, Italy; 3 Dermatology Unit, Department of Medicine and Surgery, University of Parma, Parma, Italy; 4 Skin Cancer Unit, IRCCS Istituto Romagnolo per lo Studio dei Tumori “Dino Amadori” (IRST), IRCSS, Meldola, Italy; 5 Department of Dermatology, University of Florence, Florence, Italy

**Keywords:** Basal cell carcinoma, Squamous cell carcinoma

## Abstract

Non-melanoma skin cancers (NMSC) are more frequent among men, but women (especially those aged < 40 years) have experienced steeper growth in their incidence rates in recent years. Hormonal factors were hypothesized to be playing a role in modulating NMSC risk, but the studies published to date provided conflicting results. We systematically reviewed and meta-analysed the studies focusing on the association between hormone-related characteristics (use of exogenous sex hormones, and aspects of menstrual and reproductive history) and the risk of NMSC among women. We included observational and experimental studies published in PubMed and EMBASE until February 2020. We calculated summary relative risk (SRR) and 95% confidence intervals (CI) by applying random effects models with maximum likelihood estimation, and used the I^2^ statistics to quantify the degree of heterogeneity of risk estimates across studies. Eleven independent studies encompassing a total of over 30,000 NMSC cases were included in quantitative analyses. No evidence of an increased NMSC risk emerged among ever vs. never users of oral contraceptives (SRR 1.13, 95% CI 0.88–1.45) or hormones for menopause (SRR 1.09, 95% CI 0.87–1.37). Likewise, age at menarche or at menopause and parity were not associated with NMSC risk. Heterogeneity across studies was low, and pooled results were comparable between NMSC subtypes. We found no evidence that hormonal factors play a role in the pathogenesis of NMSC among women.

## Introduction

Non-melanoma skin cancer (NMSC) refers to a heterogeneous group of cancers that originate from keratinocytes, the predominant cell type in the upper layer of the skin. The two main types of NMSC are basal cell carcinoma (BCC) and squamous cell carcinoma (SCC). NMSC are the far most common malignancy among fair-skinned population^1^, and although their prognosis is usually excellent, they impose a heavy cost burden on healthcare systems because of their high incidence and need of surgical treatment^2,3^.

NMSC recognize exposure to ultraviolet (UV) radiation (and in particular, chronic cumulative exposure, as evidenced by their strong association with older age, outdoor work, and signs of skin photodamage^4–6^) as its main behavioural risk factor, and gender disparities in epidemiology, with men being burdened with higher incidence rates than women^1^, are likely to reflect parallel differences in patterns of exposure to UV light. NMSC incidence rates are on the rise in both genders, but the observed slope appears generally steeper among women (especially those aged less than 40 years)^7–9^, and while this may imply that changes in UV exposure patterns over time are also gender-specific (e.g. concerning indoor tanning^10^), some authors have formulated the hypothesis that hormonal factors may play a role as well. Of note, this hypothesis is not without biological plausibility. For instance, it has long been known that female sex hormones may have photosensitizing properties (i.e. they may induce cutaneous disease upon exposure to UV radiation)^11^, and similarly to what is feared for other drugs^12–14^, it has been speculated that their widespread use (especially for oral contraceptives [OC]) could help accelerate the growing trends in NMSC incidence among women. Moreover, keratinocytes express estrogen receptors^15,16^, and while their function in this cell type has not been fully elucidated yet, some researchers have extended their investigations to aspects concerning the menstrual and reproductive history of women (e.g. parity, age at menarche and at menopause, etc.). However, the studies published so far that have focused on sex hormones-relate exposures in relation to NMSC risk among women have mostly provided conflicting results. In order to clarify this topic, we conducted a systematic literature review and meta-analysis of the available scientific evidence on the association between the use of exogenous sex hormones (oral contraceptives and hormone replacement therapy [HRT]) and menstrual and reproductive variables and the risk of NMSC among women.

## Results

**Figure 1:**
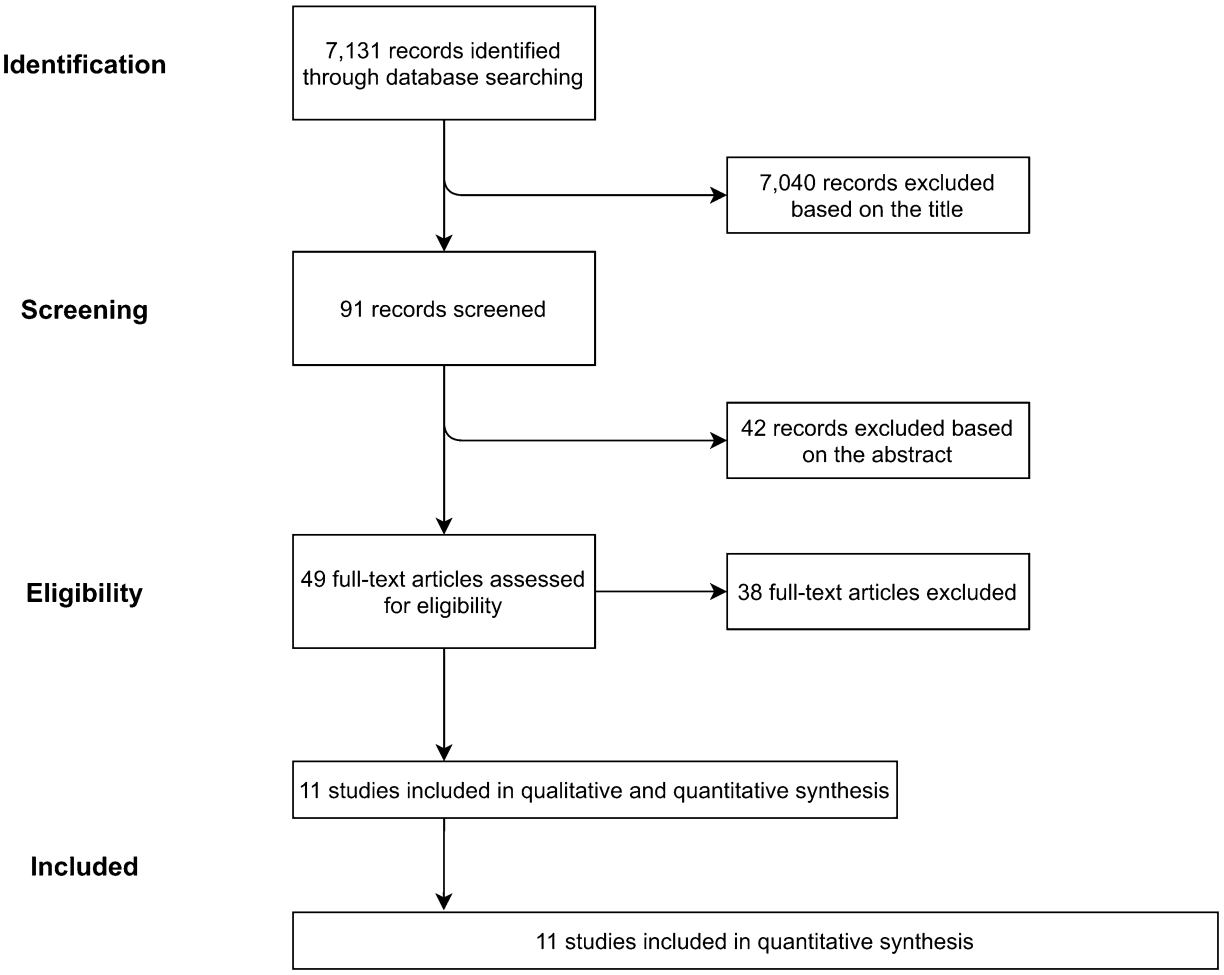
Flow-chart of the selection process for the studies included in the literature review and meta-analysis on the association between use of exogenous sex hormones, menstrual and reproductive history, and risk of non-melanoma skin cancer among women.

The literature search returned a total of 7131 non-duplicate items, of which 91 remained after being screened by title. Additional 42 papers were excluded based on their abstract (Fig. 1). The remaining 49 papers were read in full copy, of which 11 matched all inclusion criteria and were finally retained for qualitative and quantitative analyses^17–27^; no additional eligible studies were identified in their reference lists. The included studies were published between 1994 and 2018; had a cohort (n = 5), case-discordant twin cohort (n = 1), case–control (n = 3), nested case–control (n = 1), or randomized clinical trial design (n = 1); and encompassed a total of 4,926 BCC, 1,949 SCC, and 24,001 unspecified NMSC cases (over 95% of which were contributed by the study by Iversen et al.^26^) (Table 1). The exposures for which there were at least three independent risk estimates and for which, therefore, a SRR was calculated, were OC and HRT use (never vs. ever), parity, age at menarche, and age at menopause (highest vs. lowest category; no dose–response analysis was performed because of insufficient data). The methodological quality of included studies was generally high or very high, with risk of bias judges as moderate or low (Supplementary Table S1).

**Table 1:** Main characteristics of studies included in the review and meta-analysis on the association between use of exogenous sex hormones, menstrual and reproductive history, and risk of non-melanoma skin cancer among women.

Author, year	Country	Study design	Source of controls	Recruitment period	Tumour type	No. cases	No. controls	Exposures	Adjusting variables^a^
Wei, 1994^25^	USA	CC	Hospital	1987–1990	BCC	88	135	OC, HRT	Age
Milán, 2003^26^	Finland	cohort^b^	Population	1976–1999	BCC	133	133	OC	Age, socio-economic factors, UV exposure, other
Welsh, 2008^27^	USA	CC	Hospital	1993–2000	BCC	914	848	OC	Age, socio-economic factors, phenotype, UV exposure
					SCC	702	848		
Asgari, 2010^28^	USA	NCC	Population	1964–1973	SCC	516	1690	OC	Socio-economic factors, body mass index, phenotype, UV exposure, smoking, other
Tang, 2011^29^	USA	RTC	–	1993–1998	NMSC	900	900	HRT	
Birch-Johansen, 2012^30^	Denmark	Cohort	–	1993–1997	BCC	1,175	25,925	OC, HRT	Body mass index, alcohol, phenotype, alcohol
					SCC	76	25,925		
Vessey, 2013^31^	UK	Cohort	–	1968–1974	NMSC	181	16,851	OC	Age, socio-economic factors
Cahoon, 2015^32^	USA	Cohort	–	1983–2005	BCC	1,730	44,370	OC, HRT, age at menarche, parity, age at menopause	Age, birth cohort, body mass index, alcohol, UV exposure, other
Kuklinski, 2016^33^	USA	CC	Hospital	1993–2009	BCC	550	633	OC, HRT, age at menarche, parity, age at menopause	Age, phenotype
					SCC	570	746		
Iversen, 2017^34^	UK	Cohort	–	1968–1969	NMSC	22,920	23,102	OC	Age, socio-economic factors, smoking, parity
Olsen, 2018^35^	Australia	Cohort	–	2011–2014	BCC	336	10,650	OC, HRT, age at menarche, parity	Age, smoking, phenotype, UV exposure
					SCC	85	10,901		

*CC* case–control, *NCC* nested case–control, *RTC* randomized clinical trial, *BCC* basal cell cancer, *SCC* squamous cell cancer, *NMSC* non-melanoma skin cancer, *OC* oral contraceptives, *HRT* hormone replacement therapy, *UV* ultraviolet.

^a^Phenotype: any of skin, eye or hair colour, number of naevi, and phototype. UV exposure: any of intermittent or chronic exposure to natural or artificial UV radiation and/or sunburns.

^b^This is a case-discordant twin pairs cohort.

**Figure 2:**
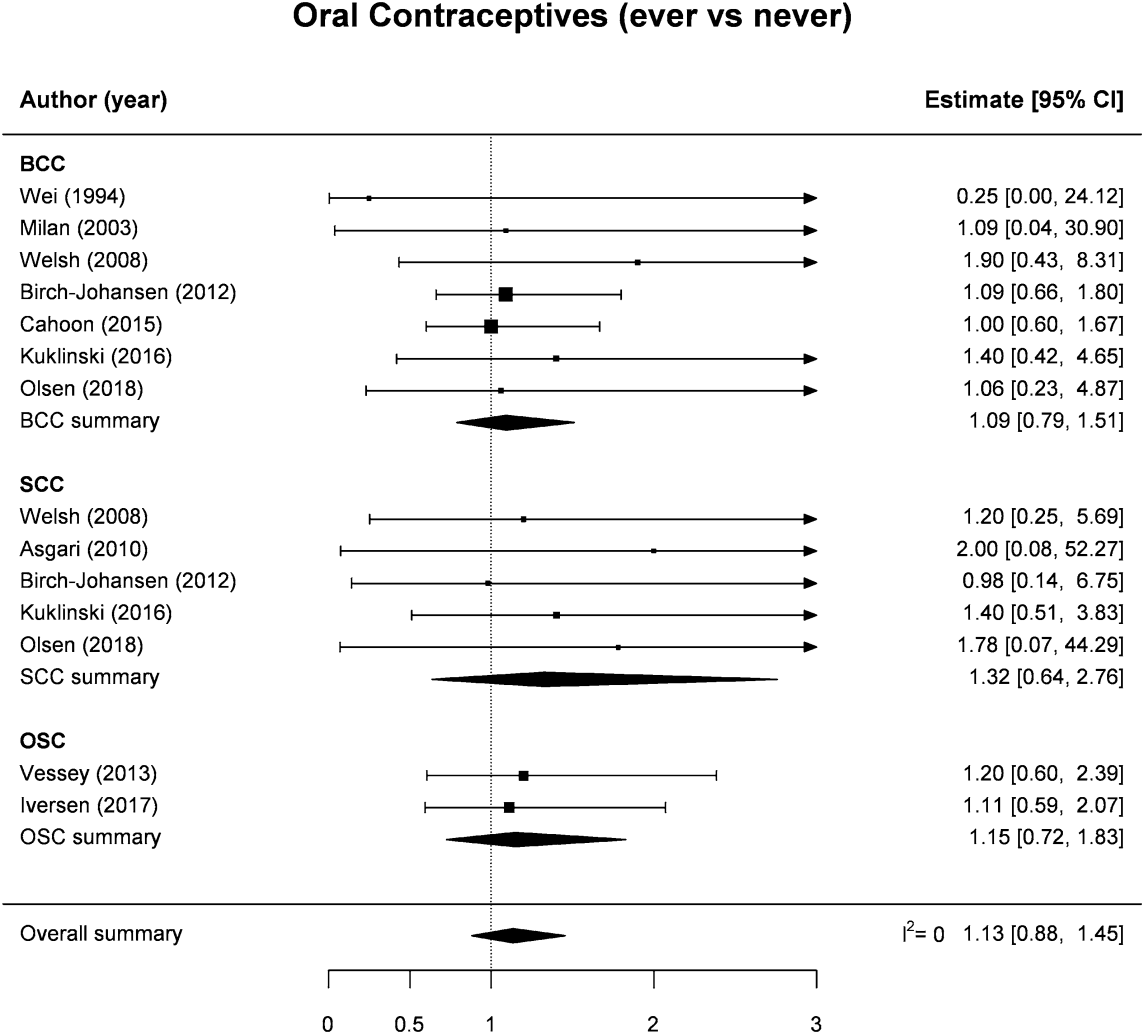
Forest plot for the association between use of oral contraceptives (ever vs. never) and the risk of non-melanoma skin cancer among women. *BCC* basal cell cancer, *SCC* squamous cell cancer, *OSC* other (not specified) non-melanoma skin cancer.

**Figure 3:**
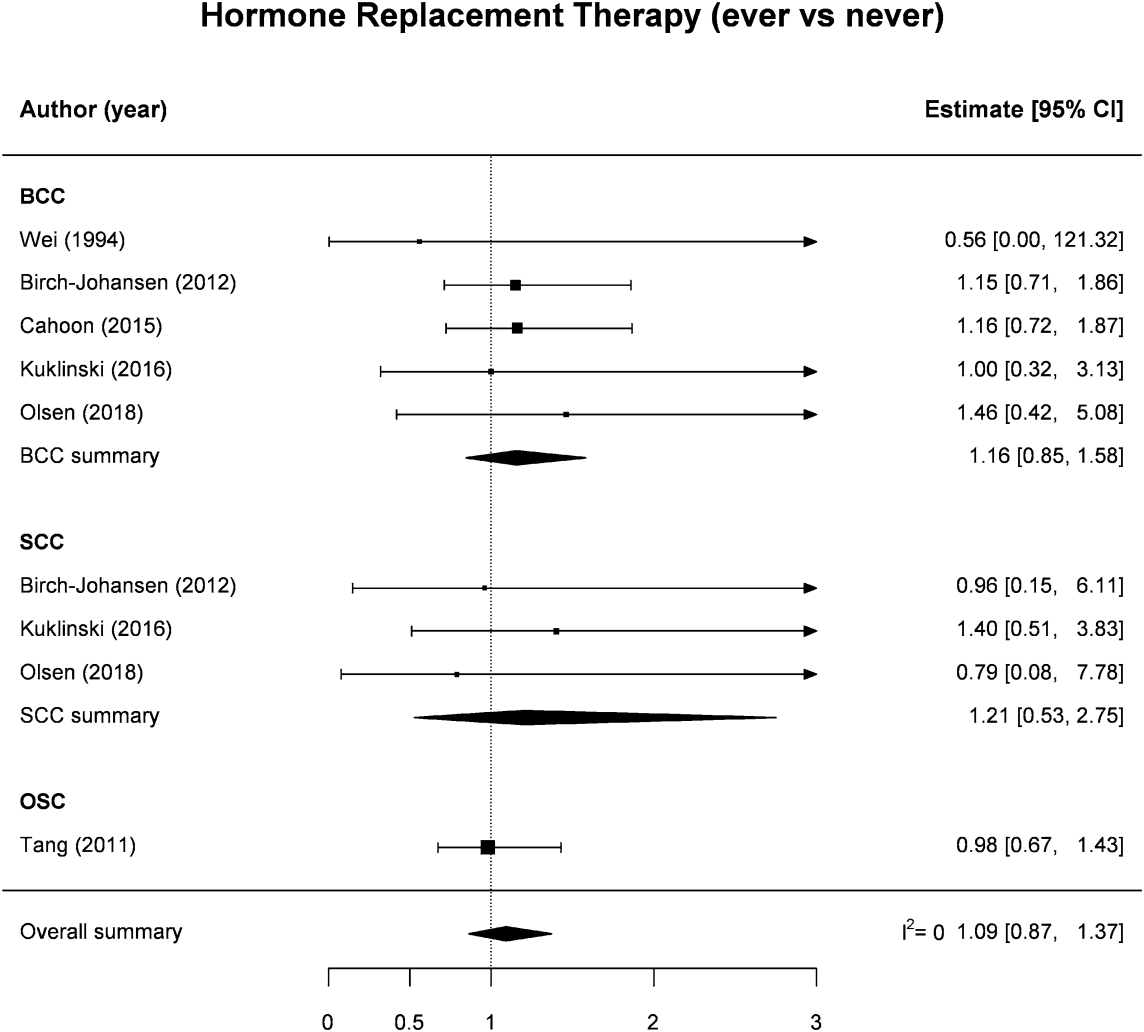
Forest plot for the association between use of hormone replacement therapy (ever vs. never) and the risk of non-melanoma skin cancer among women. *BCC* basal cell cancer, *SCC* squamous cell cancer, *OSC* other (not specified) non-melanoma skin cancer.

There were ten independent studies providing an estimate of association for the association between OC use (ever vs. never) and NMSC (Table 1, Fig. 2). In detail, four studies reported separate RRs for BCC and SCC^19,22,25,27^, three only for BCC^17,18,24^ and one study only for SCC^20^, while no distinction between NMSC subtype was made in the studies by Vessey et al. and Iversen et al.^23,26^. Pooled estimates found no evidence that NMSC risk was increased among OC ever vs. never users: the SRR was 1.13 (95% CI 0.88–1.45), with negligible heterogeneity between studies (I^2^ = 0%) and moderate, non-significant differences between BCC (1.09, 95% CI 0.79–1.51) and SCC (1.32, 95% CI 0.64–2.76) (Fig. 2). Likewise, no significant results emerged for the association between HRT use (ever vs. never) and NMSC risk: the SRR was 1.09 (95% CI 0.87–1.37, n = 9), with no evidence of heterogeneity in RR estimates across studies (I^2^ = 0%), and nearly identical pooled results for BCC (1.16, 95% CI 0.85–1.58, n = 5) and SCC (1.21, 95%CI 0.53–2.75, n = 3) (Table 1, Fig. 3).

Only three studies reported on the association between selected characteristics of the menstrual and reproductive history (namely, parity, age at menarche, and age at menopause) and the risk of NMSC among women^24,25,27^ (Table 1). As well as for the use of exogenous sex hormones, none of these variables was significantly associated with NMSC risk. In detail, the SRR for the comparison between the highest vs. lowest category of exposure was 0.89 (95%CI 0.40–1.98, I^2^ = 0%, n = 5) for parity (Supplementary Fig. S1), 1.01 (95%CI 0.47–2.18, I^2^ = 0%, n = 5) for age at menarche (Supplementary Fig. S2), and 1.22 (95%CI 0.44–3.37, I^2^ = 0%, n = 3) for age at menopause (Supplementary Fig. S3). Although the limited size did not allow conducting subgroup analyses, the visual inspection of the forest plots did not in any way suggest that an association could separately exist for either BCC or SCC.

## Discussion

We conducted a systematic literature review and meta-analyzed data from eleven independent studies encompassing a total of over 30,000 NMSC cases, and failed to find any support for the hypothesis that hormonal factors may play a role in the pathogenesis of these skin malignancies among women. Indeed, NMSC risk turned out not to be associated with either the use of exogenous sex hormones (OC and HRT) or with the menstrual and reproductive history of women (age at menarche, parity, and age at menopause). Reassuringly, while the studies included in our meta-analysis were conducted in different world areas (Europe, the USA and Australia) and spanned over more than two decades (from 1994 to 2018), the variability of findings across studies was well within what one could expect to occur by chance alone. Moreover, further robustness is provided to our conclusions by the substantial overlap of findings for the two main subtypes of NMSC, namely BCC and SCC.

The hypothesis of sex hormones being involved in NMSC pathogenesis was justified a priori by some epidemiological and laboratory data, such as the difference in NMSC incidence rates and long-term trends between genders, the known photosensitizing properties of sex hormones, and partly by the presence, although still of uncertain biological meaning, of estrogen receptors on human keratinocytes^1,7–9,11,15,16^. In contrast, some sparse experimental data from murine models suggested that endogenous estrogens may instead be protective against BCC and SCC development promoted by physical or chemical agents^28^. Regardless of biological plausibility, the possible relationship between hormonal factors and NMSC risk among women was also endowed with considerable importance from a public health perspective, given the widespread use of exogenous sex hormones (especially oral contraceptives), the globally ongoing major changes in several aspects related to the reproductive life of women, and the very high incidence rates and consequently large healthcare and social burden of NMSC. However, the present meta-analysis did not lend any support to our working hypothesis, from which it follows that the existing gender disparity in NMSC epidemiology has to be explained by resorting to alternative arguments.

The most intuitive of alternative explanations is that the reported gender differences in NMSC epidemiology are mostly the consequence of a parallel disparity between genders in terms of patterns of exposure to natural and artificial UV radiation and how these have been changing over time in recent decades. This appears reasonable as the prevalence of sunbathing and indoor tanning is socially determined and affected by gendered norms and expectations which can evolve rapidly (in addition to vary broadly geographically and across generations). In support to this view, several surveys conducted in recent years have shown how girls and young women tend to expose themselves to UV radiation more than males, despite being more aware of risks and making greater use of sunscreens^29–34^. In addition to UV exposure during leisure activities, the prevalence of occupational sunlight exposure is also largely socially determined and, therefore, likely to differ between genders and contribute to the changing NMSC incidence^35^. Finally, the gender disparity in NMSC incidence trends may by partially sustained also by the higher prevalence of self-skin examination among women reported in some (but not all) health surveys^36–38^.

To our knowledge, this is the first systematic review and meta-analysis of the scientific evidence available on the association between hormones-related characteristics and NMSC risk among women. The fair geographical distribution of the studies that were included, more than half of which had a prospective design (cohort, nested case–control or RCT), and the remarkable homogeneity of risk estimates across studies and between NMSC subtypes ensure robustness to our findings and reliability to the conclusions that can be drawn from them. The present meta-analysis has, however, also some limitations, most of which stem from the limited availability of data on some key issues, including aspects of OC and HRT use (e.g. age at first and last use, time since drug discontinuation, duration of use, and type of hormones) and variables related to the menstrual and reproductive history of women (e.g. age at first and last pregnancy and use of fertility drugs). Moreover, only a few papers adjusted their risk estimates by individual factors (e.g. body mass index or smoking habits) that are linked with the hormonal milieu among women, so that some residual confounding cannot be ruled out. Considering, however, that all variables available for the calculations pointed consistently towards the absence of any association between sex hormones-related characteristics and NMSC risk among women, we believe it unlikely that a greater amount and/or better quality of data could substantially modify the main conclusions of the present meta-analysis. Considering the low heterogeneity of risk estimates across studies despite the differences in adjusting variables, a further strength of the present review and meta-analysis lies in highlighting the poor scientific rationale supporting the need to make those adjustments.

In conclusion, we found no evidence that the risk of NMSC among women is affected by one’s menstrual and reproductive history or, more importantly, the use of exogenous sex hormones. Leisure-time UV exposure is the most important avoidable risk factor for skin cancer development: therefore, reinforcing sun awareness and promoting sun-avoidance practices remain key primary prevention measures able to favourably impact on the currently rising trends in NMSC incidence.

## Methods

This literature review and meta-analysis was planned, conducted, analyzed, and reported by adhering to the MOOSE (Meta-analysis Of Observational Studies in Epidemiology) guidelines^39^, and its study protocol was registered in the PROSPERO register of systematic reviews (registration number CRD42020161739)^40^. The literature search was conducted in the PubMed/MEDLINE (https://pubmed.ncbi.nlm.nih.gov/) and EMBASE (https://www.embase.com) bibliographic databases in September 2019 (and updated in March 2020 to cover studies published up to 2sFebruary 2020) using a search string encompassing any combination of a term denoting an exposure of interest (hormone* OR hormonal OR menarche OR menopause OR reproductive OR fertility OR parity OR pregnancy OR breastfeeding OR contraceptive* OR “replacement therapy”) AND a term denoting an outcome of interest (“basal cell cancer” OR “basal cell carcinoma” OR “squamous cell cancer” OR “squamous cell carcinoma” OR “keratinocyte skin cancer” OR “non-melanoma skin cancer” OR “nonmelanoma skin cancer”). We applied no time, geographical, or language restrictions (as long as an abstract in English was available). Two reviewers independently screened all retrieved items based on their title and abstracts: those that were considered as potentially eligible for inclusion by either reviewer were obtained and read in full copy, and their reference list was searched in order to find additional eligible papers.

Papers were eligible for inclusion that had a cohort, case–control, nested case–control, case-cohort, or randomized clinical trial design, and provided (or allowed to estimate) a measure of relative risk (RR) (i.e. incidence rate ratio, hazard ratio, risk ratio, odds ratio, or standardized incidence ratio) and a corresponding measure of statistical uncertainty (i.e. 95% confidence intervals (CI), standard errors, variance, or exact p-value) for the association between an exposure of interest (see above) and the risk of BCC, SCC, or NMSC. Ecological studies, case reports, reviews and editorials were not considered for inclusion, but their reference list were also searched to find additional eligible papers. Conference abstracts were not considered either, since they usually lack most of the information necessary for a correct interpretation of results.

The following information was extracted from each eligible paper and inputted into a dataset specifically designed for this review: publication year and country of study; study design; source, number, and gender and age distribution of study participants; average duration of follow-up (for prospective studies); whether the study had a matched design and, in case, what variables were used for matching; how the information on exposures was obtained (e.g. self-administered questionnaires, face-to-face or phone interview, medical records, etc.); and what variables were used (if any) for adjusting RR estimates. No attempt was made to contact authors to obtain missing data. We then extracted from each paper the most adjusted RR estimate (along with the available measure of statistical uncertainty) for each association of interest, and transformed into logRR and corresponding variance according to Greenland’s formula^41^. No distinction was made between the different RR measures based on the rare disease assumption. When a RR estimate for a given exposure-outcome pair was available from two or more overlapping studies, we considered that based on the largest number of NMSC cases or, in case of equal simple size, the most adjusted one. We fitted random effects models with maximum likelihood estimation to pool study-specific measures of association and obtain summary relative risk (SRR) whenever there were three or more independent RR estimates for the association between an exposure of interest and NMSC risk; corresponding 95% CI were derived assuming an underlying t distribution)^42^. We assessed whether risk estimates were homogeneous across studies by using the I^2^ statistics, which quantifies the proportion of the total variability that is attributable to actual heterogeneity rather than chance^43^. When a SRR was marked by large heterogeneity across studies (i.e. for I^2^ exceeding the 50% threshold), we used meta-regression and subgroup analyses (for continuous and categorical variables, respectively) in an attempt to find study characteristics able to explain part of the observed heterogeneity, and conducted a leave-one-out sensitivity analysis to examining how summary results changed following the exclusion of a study at a time. Meta-regression models were also fitted to assess whether pooled estimates differed between NMSC subtypes (entered as a binary variable in the model). We applied the Macaskill test to evaluate whether a publication bias against negative studies was likely to affect the summary results^44^. Finally, we rated the methodological quality of all included studies using the Newcastle–Ottawa Scale (NOS) for non-randomized studies (i.e. having a cohort, nested case–control, or case–control study design)^45^ and the revised Cochrane risk-of-bias tool for clinical trials (Rob 2.0)^46^.

Statistical analyses were conducted using SAS software, version 9.4 (SAS Institute Inc, Cary, NC, USA). All tests were two-sided and considered as statistically significant for p-values lower than 0.05.

## Supplementary Information

Supplementary Information 1.

Supplementary Information 2.

Supplementary Information 3.

Supplementary Information 4.

## Data Availability

All articles included in the present review and meta-analyses are referenced.
