# Supplementary material for: Exogenous sex hormones, menstrual and reproductive history, and risk of non-melanoma skin cancer among women: a systematic literature review and meta-analysis
Source: Sci Rep. 2021 Apr 19;11:8524. doi: 10.1038/s41598-021-88077-y (PMC8056000; doi:10.1038/s41598-021-88077-y)
Supplement: Supplementary file 1 — Supplementary Information 1. [file 41598_2021_88077_MOESM1_ESM.pdf]

Title: Exogenous sex hormones, menstrual and reproductive history, and risk of non-melanoma skin cancer among women: a systematic literature review and meta-analysis.

Authors: Saverio Caini, Simone Pietro De Angelis, Federica Corso, Carolina Fantini, Sara Raimondi, Laura Pala, Ignazio Stanganelli, Vincenzo de Giorgi, Sara Gandini

Supplementary Figure 1. Forest plot for the association between parity (high vs. low) and the risk of non-melanoma skin cancer among women.

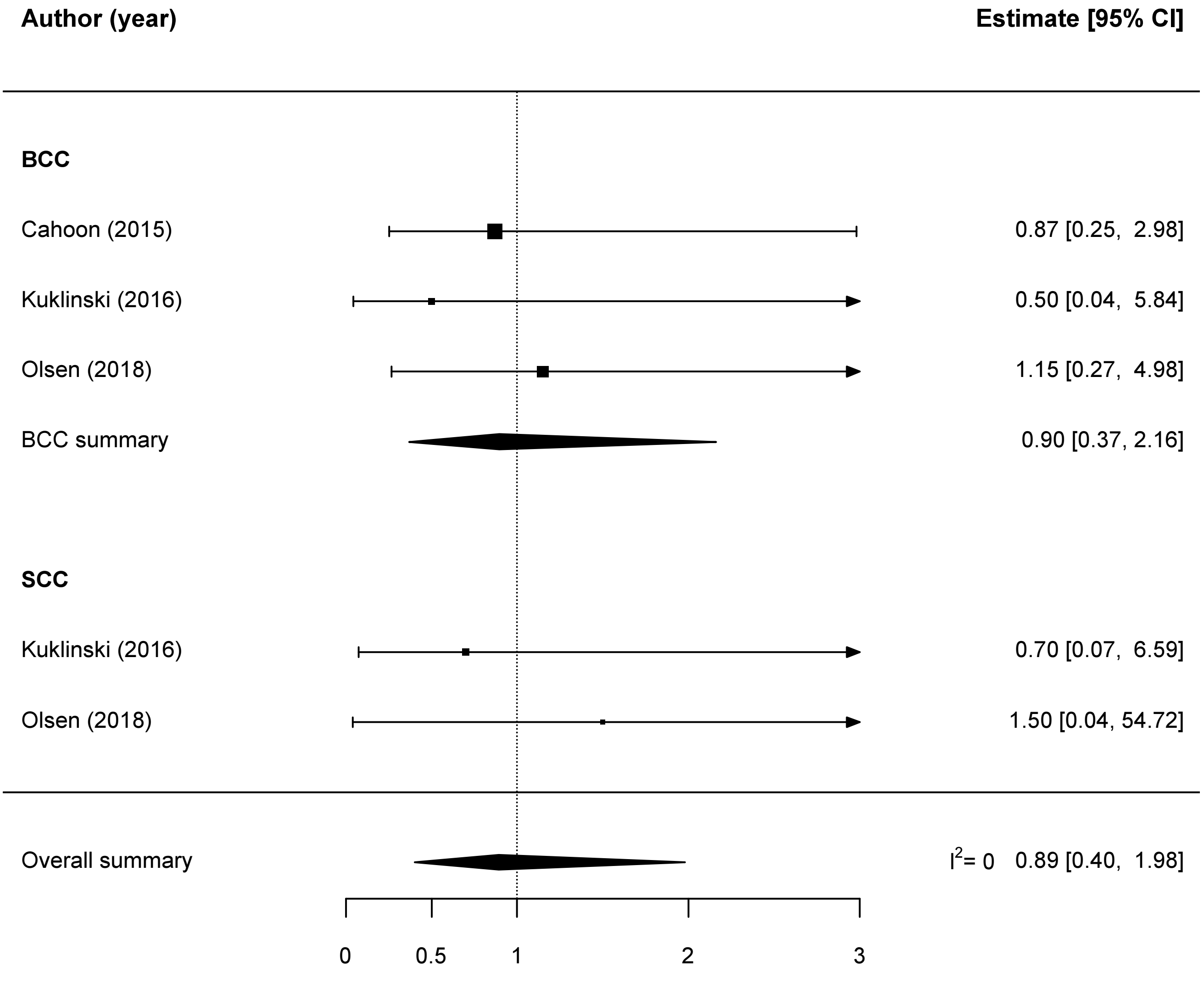

BCC: basal cell cancer. SCC: squamous cell cancer.
